# HKDC1 promotes colorectal cancer progression by regulating RCOR1 expression to activate the Wnt/β-catenin pathway, enhancing proliferation, migration, and epithelial-mesenchymal transition

**DOI:** 10.1016/j.jbc.2025.108478

**Published:** 2025-04-08

**Authors:** Shansong Huang, Qiang Pang, Yufeng Zhang, Jiaqing Cao

**Affiliations:** 1 Department of Gastrointestinal Surgery, The Second Affiliated Hospital, Jiangxi Medical College, Nanchang University, Nanchang, China; 2 Department of Oncology, Jiangxi Provincial People's Hospital, The First Affiliated Hospital of Nanchang Medical College, Nanchang, China

**Keywords:** HKDC1, RCOR1, colorectal cancer, Wnt/β-catenin pathway

## Abstract

HKDC1 (hexokinase domain containing 1) is recognized as an oncogene in various cancers, yet its role in colorectal cancer (CRC) remains unclear. This study aims to explore HKDC1 expression in CRC and its effects on tumor growth, migration, glycolysis, and EMT, as well as the underlying molecular mechanisms. Using TIMER2.0 and TCGA databases, we analyzed HKDC1 expression across multiple cancers and evaluated its prognostic value via Kaplan-Meier survival analysis. HKDC1 expression in CRC tissues was validated through western blotting, immunohistochemistry, and qRT-PCR, and its correlation with patient prognosis was assessed. Functional experiments involving HKDC1 knockdown and overexpression were performed to examine their impact on CRC cell proliferation, migration, apoptosis, and the cell cycle. Coimmunoprecipitation, immunofluorescence, and mass spectrometry identified HKDC1’s interaction with RCOR1, demonstrating its regulation of the Wnt/**β**-catenin pathway to promote CRC progression. High HKDC1 expression in CRC tissues correlated with poor patient prognosis. Knockdown of HKDC1 significantly reduced cell proliferation and migration, induced G1 phase arrest, and promoted apoptosis, whereas HKDC1 overexpression had the opposite effects. Additionally, HKDC1 promoted EMT and glycolysis through the Wnt/**β**-catenin signaling pathway. In vivo, HKDC1 knockdown inhibited tumor growth, while overexpression accelerated tumor progression. This study is the first to demonstrate that HKDC1 enhances CRC proliferation, migration, glycolysis, and EMT by modulating RCOR1 and activating the Wnt/**β**-catenin pathway. These findings suggest that HKDC1 could serve as a potential therapeutic target and prognostic marker for CRC, offering new insights for personalized treatment strategies.

Colorectal cancer (CRC) ranks among the most prevalent malignancies worldwide (1). Despite significant advancements in screening and treatment approaches in recent years, the prognosis for CRC remains poor, particularly in patients with advanced-stage CRC, where overall survival rates are still low. Therefore, identifying new therapeutic targets and prognostic biomarkers is critical in the ongoing battle against CRC.

Glucose is rapidly phosphorylated by ATP upon entering eukaryotic cells, a process catalyzed by hexokinases (HK) (2, 3, 4). Vertebrates possess four main types of hexokinases—HKI, HKII, HKIII, and HKIV—that have been thoroughly investigated. D.M. Irwin and H. Tan's study reported the identification of a novel member of the HK family, named hexokinase domain-containing protein 1 (HKDC1). Their analysis of its molecular sequence revealed that it retains the amino acid residues essential for hexokinase activity (5).

Hexokinase domain-containing protein 1 plays a crucial role in glucose metabolism, where hexokinase-dependent glucose phosphorylation is the rate-limiting step in glycolysis (5). Recent studies have identified aberrant overexpression of HKDC1 in various cancers, contributing to tumor progression (6, 7, 8). Fang *et al.* demonstrated that HKDC1 is highly expressed in gastric cancer and plays a crucial role in the pathogenesis of gastritis and gastric cancer (9). Zhang *et al.* demonstrated that HKDC1 can promote immune evasion in hepatocellular carcinoma cells (10). However, its specific role and underlying molecular mechanisms in CRC remain largely unexplored (4).

Key hallmarks of cancer progression include the remodeling of the extracellular matrix, enhanced epithelial-mesenchymal transition (EMT), and increased glycolysis (11, 12, 13). The Wnt/β-catenin signaling pathway is central to a series of developmental and disease-related processes (14). The Wnt/β-catenin signaling pathway is recognized for regulating these cancer processes and promoting tumor cell proliferation, migration, and invasion (15, 16). Investigating HKDC1's role in the Wnt/β-catenin pathway and its regulation of CRC cell metabolism and EMT is scientifically and clinically significant.

This study aimed to investigate HKDC1 expression in CRC and evaluate its effects on tumor growth, migration, and patient prognosis. Functional assays and molecular mechanism studies will clarify how HKDC1 regulates metabolic processes and EMT through the Wnt/β-catenin pathway, contributing to CRC progression. Moreover, the study will demonstrate how HKDC1 modulates RCOR1 expression to influence CRC development.

## Results

### HKDC1 is highly expressed in CRC and correlates with poor prognosis

**Figure 1:**
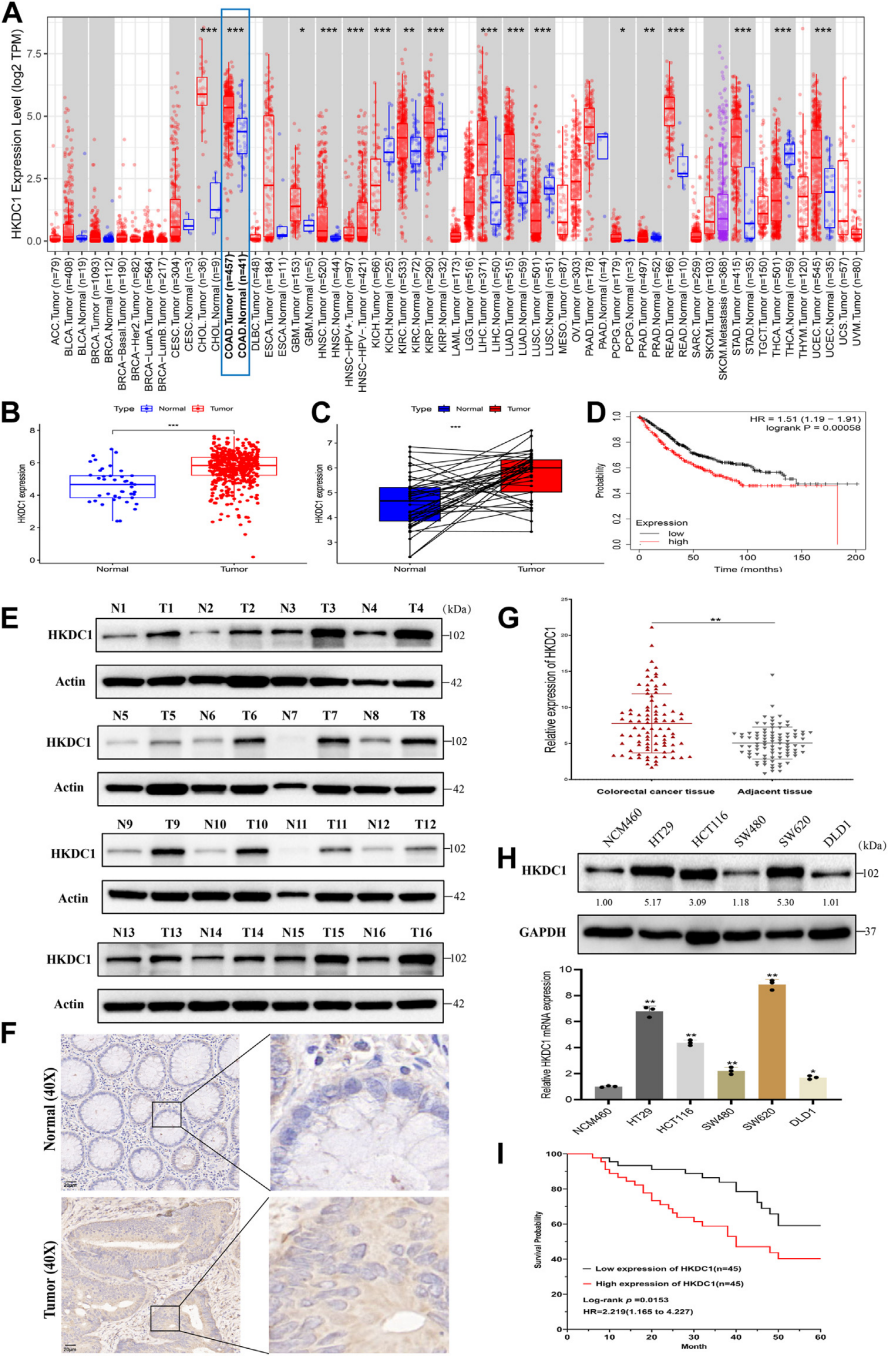
**HKDC1 is highly expressed in CRC and correlates with poor prognosis.** A, The TIMER2.0 database presents the expression profile of HKDC1 in multiple cancer types. *B*, the expression of HKDC1 in CRC. *C*, the expression of HKDC1 in paired CRC tissue samples. *D*, survival curve of HKDC1. *E–G*, Western blotting (WB), immunohistochemistry (IHC), and qRT-PCR demonstrate HKDC1 expression in both CRC and adjacent non-cancerous tissues. *H*, the expression of HKDC1 in CRC cell lines. *I*, Kaplan-Meier survival curves for 90 CRC patients from the Second Affiliated Hospital of Nanchang University. ∗*p* < 0.05; ∗∗*p* < 0.01; ∗∗∗*p* < 0.001.

Analysis using the TIMER2.0 database revealed elevated HKDC1 expression in several cancer types, notably CRC (Fig. 1*A*). Analysis of TCGA CRC data revealed significantly elevated HKDC1 expression in CRC tissues relative to normal tissues (Fig. 1, *B* and *C*). Survival analysis demonstrated that patients with high HKDC1 mRNA levels had significantly poorer overall survival (OS) compared to those with lower expression (Fig. 1*D*). Further validation through WB and IHC confirmed that HKDC1 protein levels were elevated in CRC tissues (Fig. 1, *E*–*G*). At the cellular level, we observed a similar trend of high HKDC1 expression in CRC cell lines, particularly in SW620, while DLD1 showed relatively lower expression (Fig. 1*H*). Finally, survival analysis of 90 CRC patients revealed that those with higher HKDC1 expression had significantly worse OS (Fig. 1*I*). In addition, we analyzed the relationship between HKDC1 expression and clinical traits in CRC patients and found that HKDC1 was closely associated with TNM stage, tumor size, and lymph node metastasis (Table 1 and File S2).

**Table 1:** Patient demographics and baseline characteristics

Characteristic	Expression of HKDC1			*p*-value
	Overall, N = 90^a^	Low expression of HKDC1, N = 45^a^	High expression of HKDC1, N = 45^a^	
Age				0.673^b^
≤ 60	48 (53.3%)	25 (55.6%)	23 (51.1%)	
> 60	42 (46.7%)	20 (44.4%)	22 (48.9%)	
Gender				0.206^b^
Male	46 (51.1%)	26 (57.8%)	20 (44.4%)	
Female	44 (48.9%)	19 (42.2%)	25 (55.6%)	
TNM stage				0.018^b^
Ⅰ/Ⅱ	37 (41.1%)	24 (53.3%)	13 (28.9%)	
Ⅲ/Ⅳ	53 (58.9%)	21 (46.7%)	32 (71.1%)	
Diameter of tumor(cm)				<0.001^b^
≤ 5	44 (48.9%)	30 (66.7%)	14 (31.1%)	
> 5	46 (51.1%)	15 (33.3%)	31 (68.9%)	
Lymphatic metastasis				0.031^b^
Negative	36 (40.0%)	23 (51.1%)	13 (28.9%)	
Positive	54 (60.0%)	22 (48.9%)	32 (71.1%)	
Distant metastasis				0.270^b^
Negative	74 (82.2%)	39 (86.7%)	35 (77.8%)	
Positive	16 (17.8%)	6 (13.3%)	10 (22.2%)	

a n (%).

b Pearson's Chi-squared test.

### HKDC1 promotes CRC cell growth and migration

**Figure 2:**
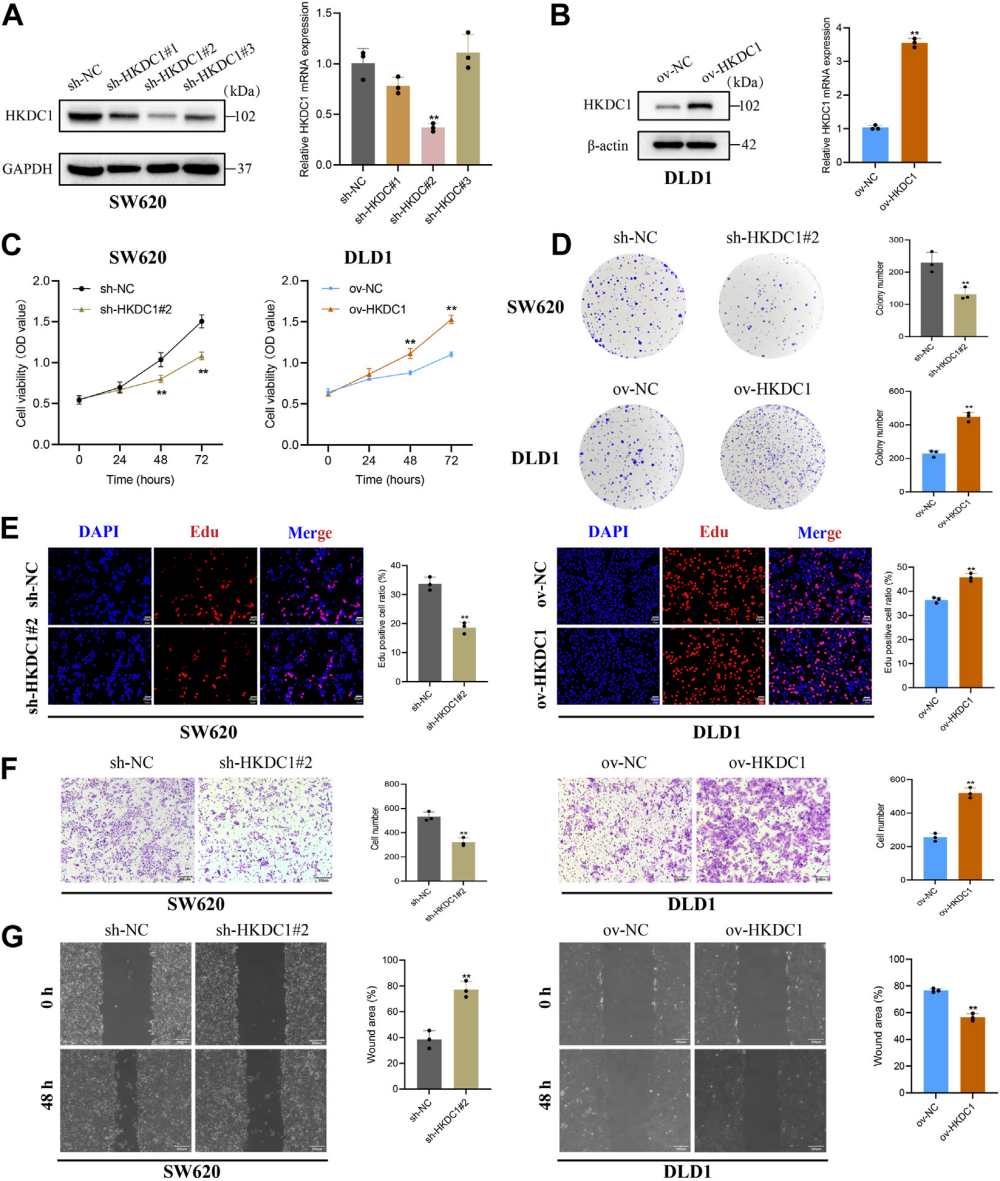
**HKDC1 promotes the proliferation and migration of CRC**. *A*, validation of HKDC1 knockdown efficiency. *B*, validation of HKDC1 overexpression efficiency. *C*, the CCK-8 assay demonstrates the effect of HKDC1 knockdown or overexpression on CRC cell proliferation. *D*, the colony formation assay shows the impact of HKDC1 knockdown or overexpression on CRC cell proliferation. *E*, the EdU assay illustrates the effects of HKDC1 knockdown or overexpression on CRC cell proliferation. *F*, the Transwell assay demonstrates the impact of HKDC1 knockdown or overexpression on CRC cell migration. *G*, the wound healing assay reveals the impact of HKDC1 knockdown or overexpression on CRC cell migration. ∗*p* < 0.05; ∗∗*p* < 0.01.

Based on HKDC1 expression levels in CRC cell lines, we selected SW620 for HKDC1 knockdown and DLD1 for HKDC1 overexpression. WB and qRT-PCR analysis confirmed that sh-HKDC1#2 was the most efficient knockdown construct for HKDC1 (Fig. 2*A*), while ov-HKDC1 exhibited significant overexpression (Fig. 2*B*). Considering the established links between tumor progression and cell proliferation, invasion, and migration, we hypothesized that HKDC1 may influence CRC cell proliferation and migration. CCK-8, EdU, and colony formation assays revealed that HKDC1 knockdown markedly suppressed SW620 cell proliferation, while HKDC1 overexpression enhanced DLD1 cell proliferation (Fig. 2, *C*–*E*). Transwell migration and wound healing assays demonstrated that HKDC1 knockdown significantly decreased SW620 cell migration, whereas HKDC1 overexpression increased DLD1 cell migration (Fig. 2, *F* and *G*).

### HKDC1 promotes CRC cell cycle progression and inhibits apoptosis

**Figure 3:**
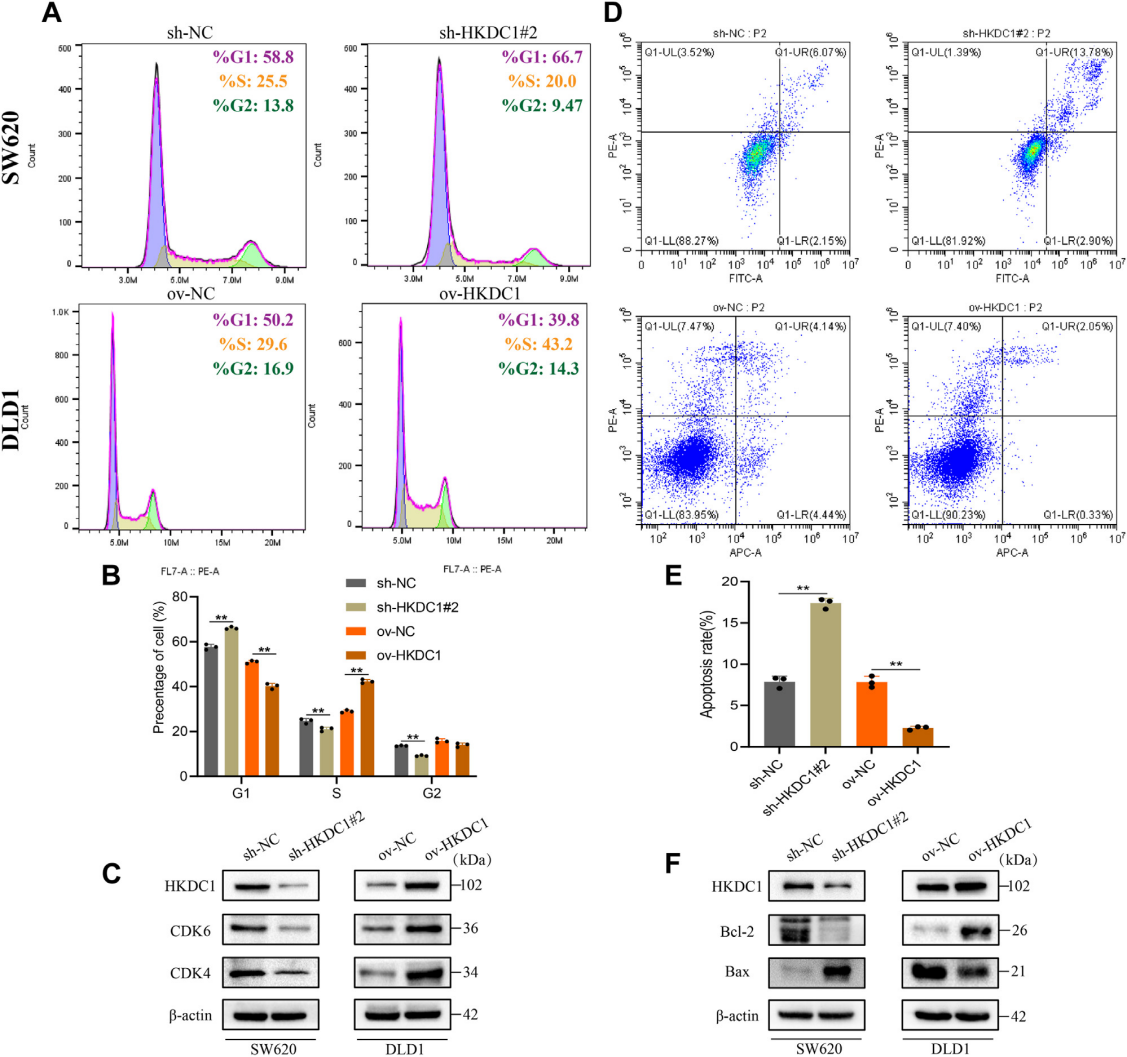
**HKDC1 facilitates cell cycle progression and suppresses apoptosis in CRC**. *A* and *B*, The effect of HKDC1 knockdown or overexpression on the CRC cell cycle. *C*, WB analysis of cell cycle-related proteins. *D* and *E*, The effect of HKDC1 knockdown or overexpression on CRC cell apoptosis. *F*, WB analysis of proteins associated with apoptosis. ∗*p* < 0.05; ∗∗*p* < 0.01.

Given the close association between cell cycle dysregulation and tumor cell proliferation and the counteracting role of apoptosis, we investigated the potential relationship between HKDC1, cell cycle progression, and apoptosis in CRC cells. Flow cytometry analysis revealed that HKDC1 knockdown in SW620 cells significantly elevated the proportion of cells in the G1 phase (Fig. 3, *A* and *B*). Additionally, HKDC1 knockdown significantly increased cell apoptosis (Fig. 3, *D* and *E*). In contrast, overexpression of HKDC1 in DLD1 cells produced the opposite effects. WB analysis further confirmed that in the HKDC1 knockdown group, CDK4, CDK6, and Bcl-2 were decreased, while Bax was upregulated (Fig. 3, *C*/*F*). Overexpression of HKDC1 yielded the reverse pattern (Fig. 3, *C*/*F*).

### HKDC1 promotes EMT and enhances glycolysis *via* the Wnt/**β**-catenin pathway in CRC

**Figure 4:**
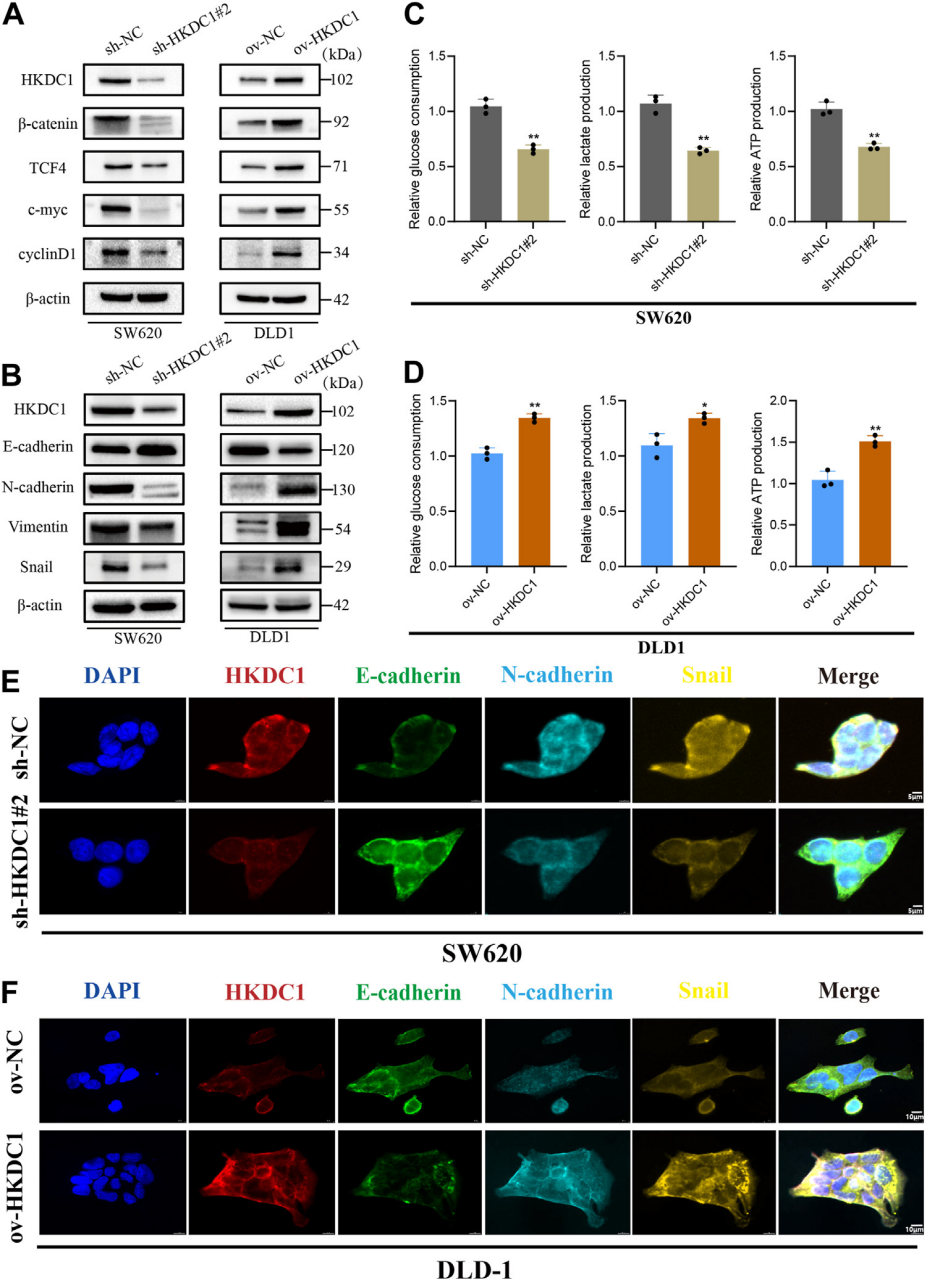
**HKDC1 enhances the Wnt/β-catenin signaling pathway, EMT, and glycolysis in CRC cells**. *A*, WB analysis of HKDC1 knockdown or overexpression effects on the Wnt/β-catenin pathway. *B*, WB analysis of EMT-related proteins. *C* and *D*, the impact of HKDC1 on glucose consumption, lactate production, and ATP generation in CRC cells. ∗*p* < 0.05; ∗∗*p* < 0.01. *E* and *F*, immunofluorescence results demonstrate the impact of HKDC1 regulation on the expression of EMT pathway-related proteins.

The Wnt/β-catenin pathway is pivotal in cancer progression, especially in facilitating EMT. We hypothesized that HKDC1 may regulate CRC progression *via* the Wnt/β-catenin pathway, thereby promoting EMT. WB analysis validated the hypothesis: HKDC1 knockdown decreased β-catenin, TCF4, c-Myc, and Cyclin D1 expression, whereas HKDC1 overexpression reversed these changes (Fig. 4*A*). HKDC1 knockdown significantly decreased N-cadherin, vimentin, and Snail levels, while increasing E-cadherin expression. Overexpression of HKDC1 produced the opposite results (Fig. 4*B*). Immunofluorescence assay further confirmed the effect of HKDC1 regulation on the expression of EMT pathway proteins (Fig. 4, *E* and *F*).

As HKDC1 belongs to the hexokinase family, we investigated its influence on glycolysis. Glucose consumption, lactate production, and ATP generation were measured to assess glycolysis. HKDC1 knockdown in SW620 cells significantly decreased glucose consumption, lactate production, and ATP generation relative to control cells (Fig. 4*C*). Conversely, overexpression of HKDC1 in DLD1 cells increased these glycolytic indicators (Fig. 4*D*).

### HKDC1 promotes CRC growth

**Figure 5:**
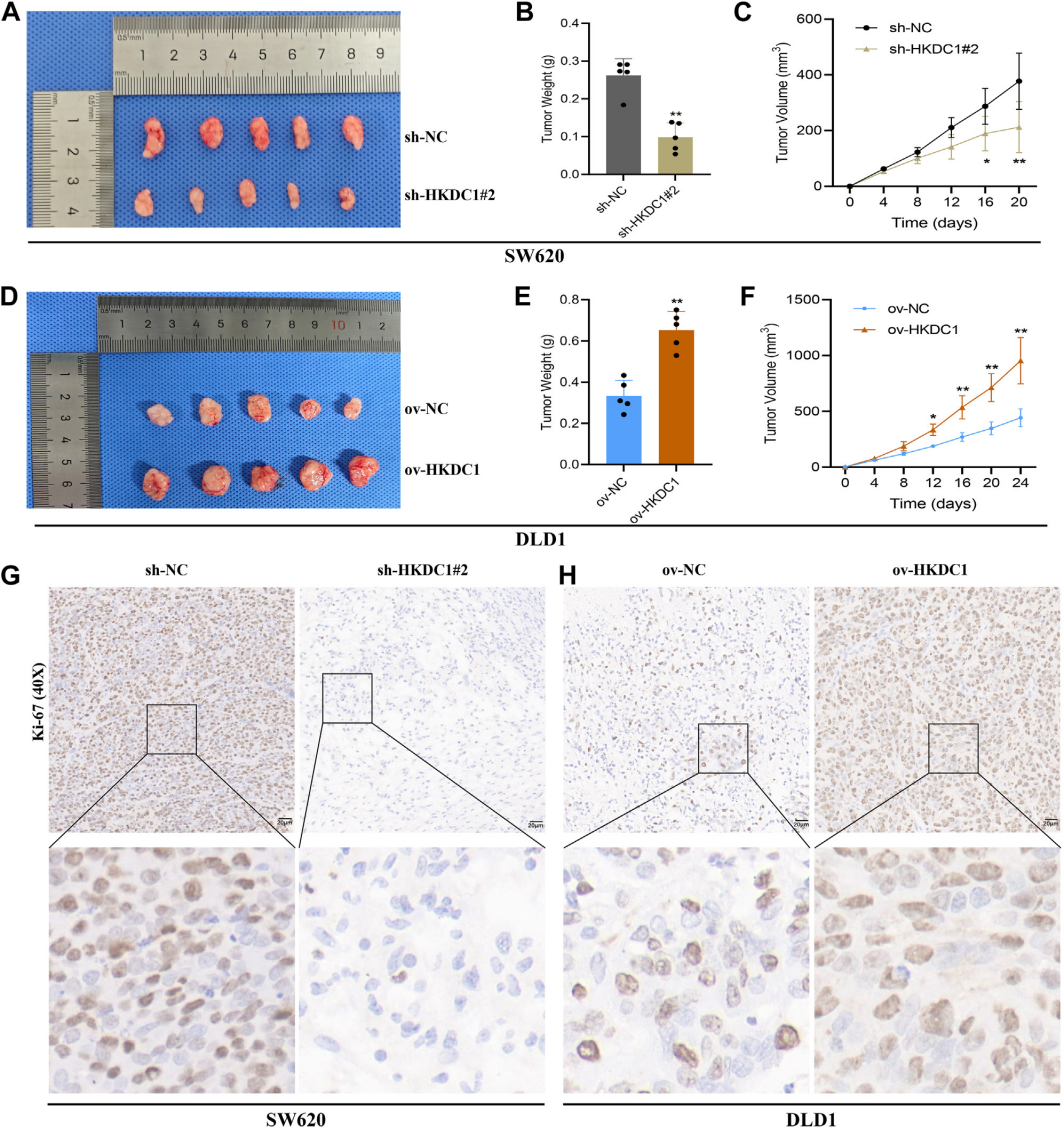
**HKDC1 promotes the *in vivo* growth of CRC cells**. *A*, The effect of HKDC1 knockdown on tumor development in nude mice. *B*, impact of HKDC1 knockdown on tumor weight in nude mice. *C*, the effect of HKDC1 knockdown on tumor growth in nude mice. *D*, the effect of HKDC1 overexpression on tumor development in nude mice. *E*, impact of HKDC1 overexpression on tumor weight in nude mice. *F*, the effect of HKDC1 overexpression on tumor growth in nude mice. *G* and *H*, IHC analysis shows the effect of HKDC1 knockdown or overexpression on Ki-67 expression in CRC cells. ∗*p* < 0.05; ∗∗*p* < 0.01.

To assess the role of HKDC1 in CRC growth, SW620 cells (sh-NC and sh-HKDC1#2) and DLD1 cells (ov-NC and ov-HKDC1) were subcutaneously injected into nude mice. The sh-HKDC1#2 group exhibited significantly smaller tumor volume and size compared to the sh-NC group (Fig. 5, *A* and *B*), and the growth rate was notably slower (Fig. 5*C*). IHC analysis indicated reduced HKDC1 and Ki-67 expression levels in the sh-HKDC1#2 group relative to the sh-NC group (Fig. 5*G*). Tumors in the ov-HKDC1 group exhibited larger size and faster growth compared to those in the ov-NC group (Fig. 5, *D*–*F*), with higher HKDC1 and Ki-67 expression (Fig. 5*H*). These results demonstrate that HKDC1 promotes CRC growth *in vivo*.

### HKDC1 promotes CRC growth and migration through RCOR1

**Figure 6:**
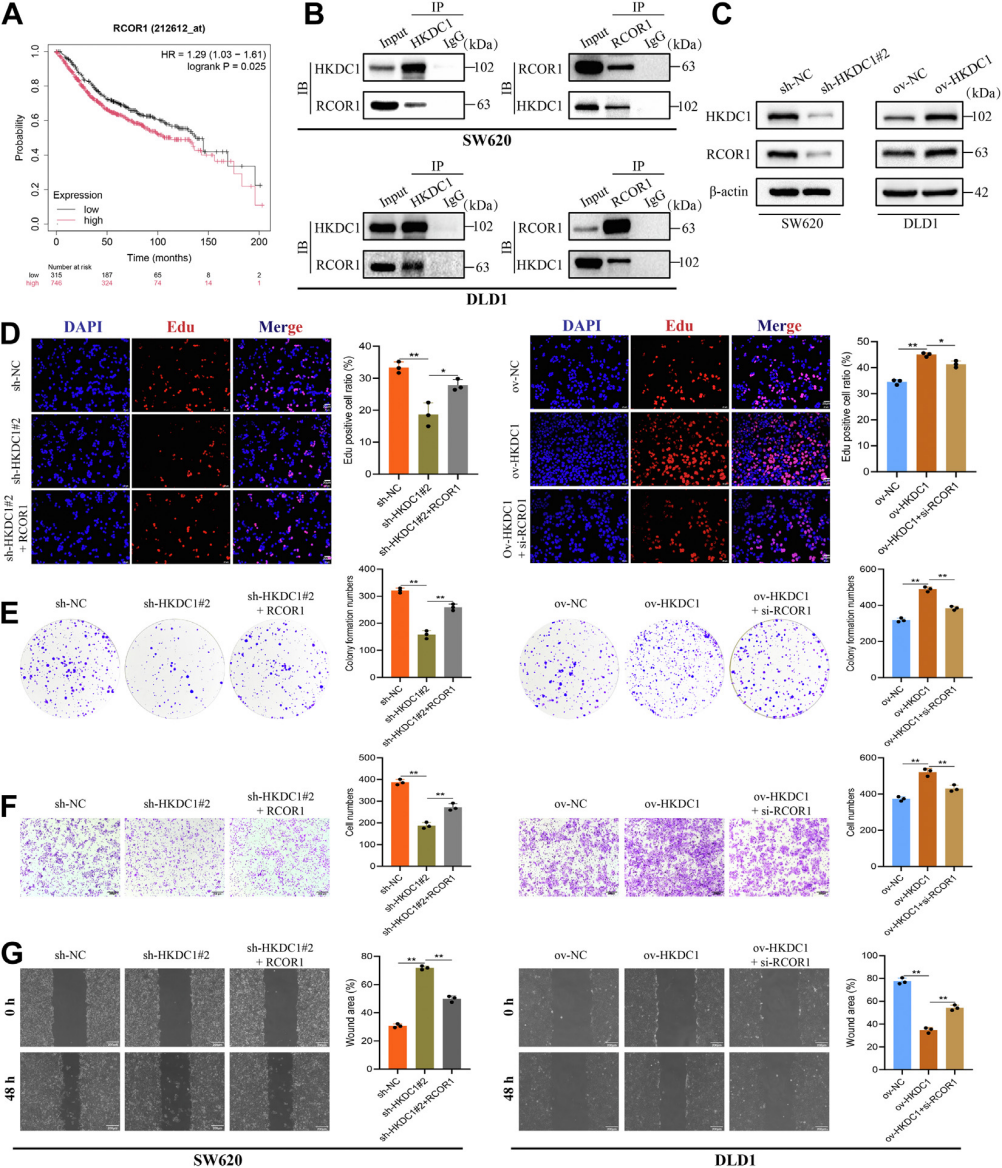
**HKDC1 regulates RCOR1 to promote the proliferation and migration of CRC cells**. *A*, survival analysis of RCOR1. *B*, co-immunoprecipitation (Co-IP) analysis of HKDC1 and RCOR1 interaction. *C*, WB analysis of the regulatory relationship between HKDC1 and RCOR1. *D* and *E*, EdU and colony formation assays comparing the proliferation capacity of various CRC cell groups. *F* and *G*, Transwell and wound healing assays were conducted to compare the migration capacities of various CRC cell groups. ∗*p* < 0.05; ∗∗*p* < 0.01.

To further investigate the mechanisms by which HKDC1 promotes CRC progression, we performed Co-IP and mass spectrometry analyses, identifying RCOR1 as an interacting partner of HKDC1 (Supplementary File 1). Survival analysis showed that high RCOR1 expression was associated with poorer OS in CRC patients (Fig. 6*A*). WB confirmed the interaction between HKDC1 and RCOR1 (Fig. 6*B*). Additionally, we found that HKDC1 knockdown reduced RCOR1 expression, while HKDC1 overexpression increased RCOR1 expression (Fig. 6*C*). These results suggest that RCOR1 mediates HKDC1's promotion of CRC progression. RCOR1 overexpression counteracted the suppression of CRC cell proliferation and migration caused by HKDC1 knockdown, while RCOR1 knockdown inhibited the tumor-promoting effects of HKDC1 overexpression (Fig. 6, *D*–*G*). HKDC1 enhances CRC cell proliferation and migration through RCOR1 upregulation.

### HKDC1 regulates the CRC cell cycle, apoptosis, and EMT through RCOR1 *via* the Wnt/**β**-catenin pathway

**Figure 7:**
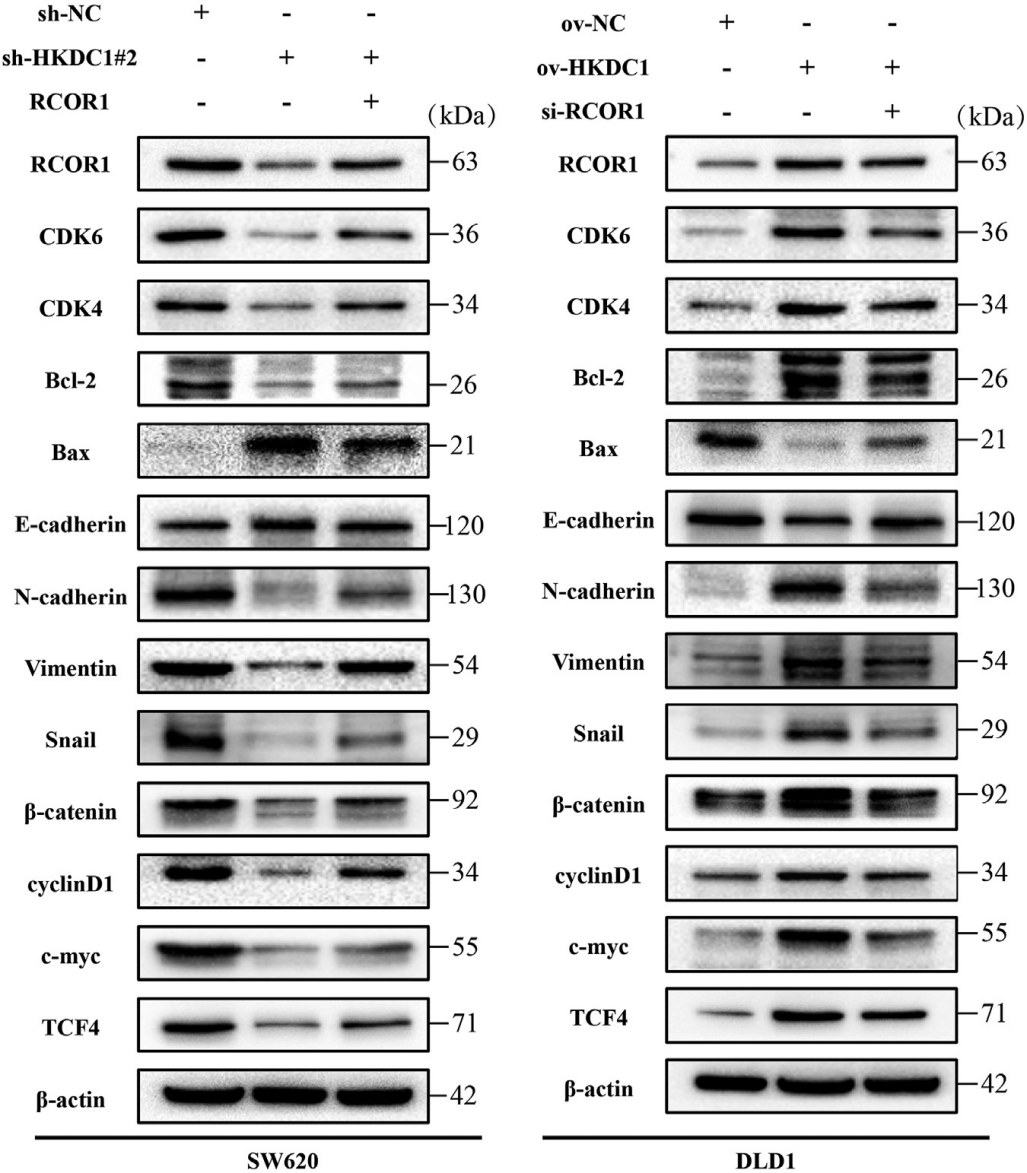
**HKDC1 affects CRC cell cycle, apoptosis, and EMT *via* RCOR1 and activates the Wnt/β-catenin pathway**.

**Figure 8:**
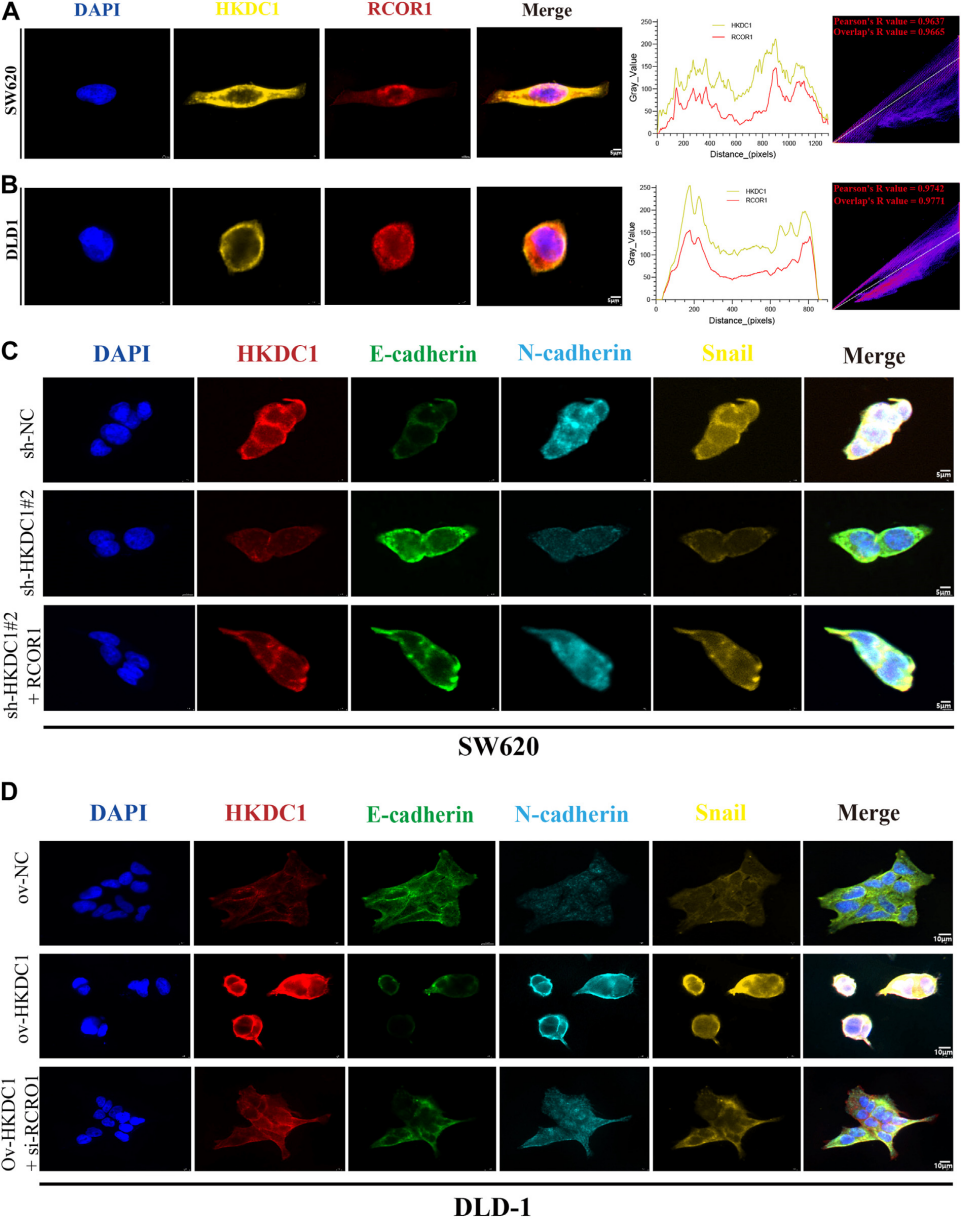
**Immunofluorescence confirmed the interaction between HKDC1 and RCOR1, as well as the effect of HKDC1 regulation on the expression of EMT pathway proteins.***A* and *B*, the colocalization between HKDC1 and DLD-1 was visualized by a confocal laser scanning microscope in different cell lines. *C* and *D*, Immunofluorescence results demonstrate the impact of HKDC1 regulation and rescue experiment on the expression of EMT pathway-related proteins.

We investigated if HKDC1 influences the CRC cell cycle, apoptosis, and EMT through RCOR1 *via* the Wnt/β-catenin pathway using WB analysis (Fig. 7). RCOR1 overexpression in the HKDC1 knockdown context significantly increased CDK4, CDK6, Bax, N-cadherin, vimentin, β-catenin, TCF4, c-Myc, and Cyclin D1, while decreasing Bcl-2 and E-cadherin. RCOR1 knockdown in the context of HKDC1 overexpression decreased the expression of CDK4, CDK6, Bax, N-cadherin, vimentin, Snail, β-catenin, TCF4, c-Myc, and Cyclin D1, while increasing Bcl-2 and E-cadherin levels. These findings indicate that HKDC1 influences CRC progression *via* RCOR1 and the Wnt/β-catenin pathway, enhancing cell cycle progression and EMT while suppressing apoptosis. Confocal microscopy results also revealed the binding between HKDC1 and RCOR1 in colon cancer cells (Fig. 8, *A* and *B*). Through further immunofluorescence staining experiments, we confirmed the impact of HKDC1 regulation and rescue on EMT pathway-related protein expression (Fig. 8, *C* and *D*).

**Figure 9:**
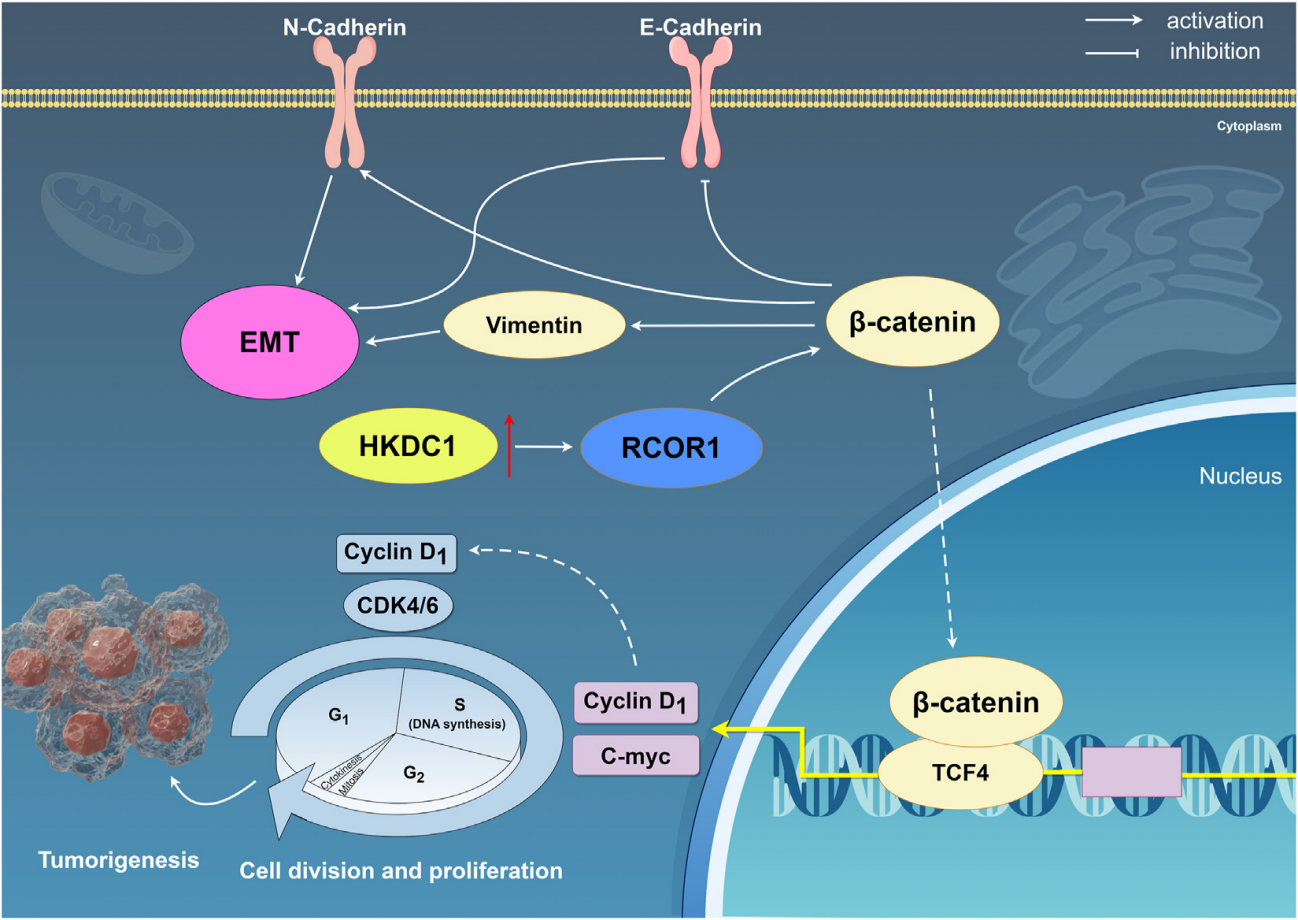
**Schematic diagram of the regulatory mechanisms by which HKDC1 regulates CRC proliferation, apoptosis, and EMT**.

In summary, our study demonstrates that HKDC1 interacts with RCOR1 and upregulates its expression, thereby promoting CRC cell cycle progression and suppressing apoptosis while enhancing EMT. A schematic diagram illustrating the mechanism by which HKDC1 promotes CRC progression was created using Figdraw (Fig. 9).

## Discussion

CRC is a common malignancy in the digestive system. This underscores the urgent need for effective therapeutic targets to mitigate its effects. Our study demonstrated that HKDC1 significantly promotes CRC cell proliferation and migration, as evidenced by both *in vitro* and *in vivo* experiments.

Increasing evidence suggests that HKDC1 contributes to the progression of various cancers. For example, Wang *et al.* HKDC1 promotes tumor progression and glycolysis in lung adenocarcinoma *via* the AMPK/mTOR pathway (17). Similarly, Khan *et al.* demonstrated that silencing HKDC1 drives metabolic reprogramming from the TCA cycle to mitochondrial pathways, inhibiting hepatocellular carcinoma (HCC) development (18). Moreover, Wang *et al.* found that HKDC1 is overexpressed in gastric cancer, leading to resistance against cisplatin, oxaliplatin, and 5-fluorouracil (19). In HCC, HKDC1 has also been shown to enhance immune evasion by promoting PD-L1 expression (10). Pang *et al.* revealed that HKDC1 overexpression is associated with pancreatic cancer progression and prognosis (20). Despite these findings, there has been little investigation into the role of HKDC1 in CRC. Our research observed elevated HKDC1 expression in CRC. Knockdown of HKDC1 significantly inhibited CRC cell proliferation and migration. Flow cytometry analysis confirmed that HKDC1 knockdown induced G2 cell cycle arrest and apoptosis. We demonstrated that HKDC1 promotes glycolysis in CRC, an effect reversed by its overexpression.

REST Corepressor 1 (RCOR1) is a key protein involved in gene expression regulation, particularly in the transcriptional repression of neuronal genes (21, 22). By interacting with REST and other cofactors, RCOR1 modulates chromatin structure to inhibit gene transcription, playing a vital role in processes such as neurodevelopment, cellular differentiation, and cancer progression (23, 24, 25, 26). Although RCOR1 has been implicated in tumorigenesis across various cancers (27, 28), its role in CRC remains unexplored. Through Co-IP and mass spectrometry, we identified an interaction between HKDC1 and RCOR1, demonstrating that HKDC1 regulates RCOR1 protein expression. To validate RCOR1's contribution to HKDC1-mediated oncogenesis, we constructed RCOR1 knockdown and overexpression models. Overexpression of RCOR1 significantly reversed the inhibitory effects of HKDC1 knockdown on CRC proliferation and migration, while silencing RCOR1 mitigated HKDC1's pro-tumor effects.

Wnt/β-catenin pathway abnormalities are well-established in numerous cancers, particularly in CRC (29, 30, 31). This pathway is essential for tumorigenesis by stimulating cell proliferation and inhibiting apoptosis (32, 33). Our investigation revealed that HKDC1 knockdown diminishes the expression of crucial Wnt/β-catenin signaling proteins, such as β-catenin, TCF4, c-myc, and cyclinD1. HKDC1 overexpression upregulated these proteins, indicating that HKDC1 activates the Wnt/β-catenin pathway to promote CRC progression. Emerging evidence indicates that the Wnt/β-catenin pathway promotes EMT (34, 35, 36). We examined the role of HKDC1 in regulating EMT through Wnt/β-catenin signaling. Our results suggest that knockdown of HKDC1 decreases N-cadherin, vimentin, and increases E-cadherin, while overexpression of HKDC1 produces the opposite effect. To further examine RCOR1's role in this process, we demonstrated that RCOR1 overexpression mitigated the inhibitory effects of HKDC1 knockdown on EMT and Wnt/β-catenin signaling. Immunofluorescence experiments further validated the aforementioned results.RCOR1 knockdown in HKDC1-overexpressing cells diminished EMT and Wnt/β-catenin signaling promotion. Through colocalization and immunoprecipitation (IP) experiments, we demonstrated the interaction between HKDC1 and RCOR1 in colon cancer cells. These findings provide robust evidence of the molecular interaction between HKDC1 and RCOR1, shedding light on their potential relationship within the tumor microenvironment of colon cancer.

To further confirm HKDC1's role in CRC progression, we conducted *in vivo* xenograft experiments. Knockdown of HKDC1 significantly suppressed tumor growth, whereas overexpression accelerated it. Immunohistochemistry (IHC) analysis revealed that Ki-67, a cell proliferation marker, decreased with HKDC1 knockdown, and increased with HKDC1 overexpression.

While our study provides new insights into HKDC1's role in promoting CRC through RCOR1, there are still limitations that warrant further investigation. Although we confirmed that HKDC1 promotes CRC progression through xenograft models, its role in CRC metastasis has not yet been validated *in vivo*. Moreover, although we demonstrated that HKDC1 regulates RCOR1 expression, the exact mechanisms, including potential post-translational modifications such as ubiquitination or lactylation, remain unclear and require further exploration.

Our study concludes that HKDC1 enhances CRC cell proliferation, migration, and EMT *via* RCOR1-mediated activation of the Wnt/β-catenin signaling pathway while inhibiting apoptosis. These findings indicate that HKDC1 may be a viable therapeutic target for CRC treatment.

## Experimental procedures

### Bioinformatics analysis

The TIMER2.0 database was used to analyze HKDC1 expression across various cancer types. Transcriptomic and clinical data from the TCGA-COAD dataset were obtained from the TCGA database for analysis.

### Clinical tissue samples

CRC tissue samples were collected from patients at the Second Affiliated Hospital of Nanchang University. No patients had undergone radiotherapy or chemotherapy prior to surgery. Tissue samples were preserved in 4% paraformaldehyde (Solarbio Science &Technology Co, Ltd, Beijing, China) for further analysis. The Ethics Committee of the Second Affiliated Hospital of Nanchang University approved the study, and all patients provided written informed consent. The human research covered by the study adheres strictly to the Declaration of Helsinki principles.

### Cell culture and transfection

Human CRC cell lines were purchased from the Shanghai Institute of Cell Research, Chinese Academy of Sciences. The normal colorectal cell line NCM460 and colorectal cancer cell line HCT-116 were cultured in RPMI-1640 medium (Solarbio Science & Technology Co, Ltd) with 10% fetal bovine serum (FBS, ExCell Bio). Colorectal cancer cell lines from different species, including HT-29, HCT-116, SW-480, SW-620, and DLD-1, were cultured in DMEM medium (Solarbio Science & Technology Co, Ltd) supplemented with 10% fetal bovine serum. All cell lines were incubated in a constant temperature incubator set at 37 °C and with a CO2 concentration of 5%.

Lentiviral shRNAs targeting HKDC1 (sh-HKDC1#1, sh-HKDC1#2, sh-HKDC1#3), control shRNA (sh-NC), overexpression plasmids (ov-HKDC1), and control vectors (ov-NC) were obtained from General-Biol. RCOR1 plasmids and siRNA were sourced from Hanbio, Shanghai, China. Lentiviral transfection followed the manufacturer's instructions. Lipofectamine 3000 (Thermo Fisher Scientific) was used for plasmid and siRNA transfection.

### Quantitative real-time PCR (qRT-PCR) and Western Blotting (WB)

Total RNA was extracted with Trizol reagent and reverse-transcribed into cDNA (Takara, RR047A). SYBR Green Master Mix (Takara, RR420A) was used for real-time PCR. The primers used for qPCR were HKDC1 forward (5′-ATCCTGGCAAGCAGAGA-3′) and reverse (5′-ATCCTGGCAAGCAGAGATACG-3'), with GAPDH serving as the internal control.

Total protein for Western blotting was extracted from NCM-460, SW-620, SW-480, HT-29, DLD-1, and HCT-116 cells using RIPA lysis buffer(Solarbio Science &Technology Co, Ltd). Proteins were subjected to SDS-PAGE and subsequently transferred to PVDF membranes(Merck Millipore Ltd). Membranes were blocked with 5% non-fat milk (Beyotime Biotechnology) for 2 h and incubated overnight with primary antibodies: HKDC1 (1:1000, Abcam), RCOR1 (1:1000, Proteintech), Bax (1:2000, Proteintech), Bcl-2 (1:2500, Proteintech), CDK4 (1:1000, Proteintech), CDK6 (1:2000, Proteintech), Cyclin D1 (1:5000, Proteintech), β-catenin(1:5000, Proteintech), TCF4 (1:2000, Proteintech), c-Myc(1:2000, Proteintech), N-cadherin (1:5000, Proteintech), E-cadherin (1:50,000, Proteintech), and vimentin (1:1000, Proteintech). HRP-conjugated secondary antibodies (UElandy) were used for detection, and immunoblots were visualized and analyzed.

### Cell proliferation assays

Transfected cells were seeded at 5000 cells per well in 96-well plates (JET BIOFIL, Guangzhou, China), with CCK-8 reagent (UElandy) added at 0, 24, 48, and 72 h. Absorbance at 450 nm was measured to assess proliferation.

DNA synthesis was detected using an EdU assay kit (UElandy) following the manufacturer's instructions. Fluorescence microscopy was used to observe EdU-positive cells.

In the colony formation assay, transfected cells (1000 cells/well) were seeded into 6-well plates (JET BIOFIL) and cultured for 2 weeks. Cells were fixed with 4% paraformaldehyde, stained with Crystal violet(Solarbio Science &Technology Co, Ltd), and colonies were counted.

### Cell migration assays

Transfected cells (30,000 cells/well) were placed in 24-well plates(JET BIOFIL), the upper chamber with serum-free medium, while the lower chamber contained medium with 20% fetal bovine serum. After 48 h, cells were fixed, stained, and counted post-migration.

Transfected cells were cultured to 90% confluence in 6-well plates for the wound healing assay. A wound was made by a 200 μl pipette tip, and cells were cultured in a serum-free medium. Wound closure was observed at 0 and 48 h under a microscope.

### Cell cycle and apoptosis analysis

Apoptosis Analysis: Transfected cells were stained with Annexin V-FITC and PI (UElandy) following the manufacturer's instructions and analyzed *via* flow cytometry.

Cell Cycle Analysis: Cells were fixed in 75% ethanol and incubated with PI staining solution (UElandy) for 30 min. Cell cycle distribution was assessed using flow cytometry.

### Co-immunoprecipitation (Co-IP)

SW620 and DLD1 cells were lysed using pre-chilled RIPA lysis buffer containing PMSF(Solarbio Science &Technology Co, Ltd). Cell lysates were incubated with anti-HKDC1 or anti-RCOR1 antibodies, then with Protein A/G Magnetic Beads (Selleck Chemicals). The precipitates were collected, washed, and subjected to SDS-PAGE for further analysis.

### Glycolysis assays

Supernatants from transfected cells were collected, and glucose and lactate levels were measured using respective kits (Beyotime Biotechnology). ATP production was measured in cell lysates using an ATP assay kit (Beyotime Biotechnology).

### Xenograft tumor model

Twenty four-week-old male BALB/c nude mice (17–22g) were obtained from Beijing SPF Biotechnology Co., Ltd. Each mouse was subcutaneously injected with 5 × 10ˆ6 cells from one of the following groups: sh-NC, sh-HKDC1#2, ov-NC, or ov-HKDC1. Tumor dimensions were measured every 4 days post-injection using a caliper. The tumor volume was calculated using the formula: Volume (mm^3^) = Length (mm) × Width (mm)^2^ × 0.52. After 20 days, mice were euthanized, and tumor tissues were excised and weighed. All animal experiments were approved by the Animal Ethics Committee of Nanchang University.

### Immunohistochemistry

Paraffin-embedded tumors and adjacent normal tissues from patients, as well as xenograft tumors, were sectioned and incubated with anti-HKDC1 or anti-Ki-67 antibodies. DAB was utilized for visualization, and images were captured microscopically.

### Immunofluorescence

Colon cancer cells from different treatment groups were seeded at a density of 1 × 10∧^5^ cells per well in a 24-well plate and incubated at 37 °C for 24 to 36 h. Once the cells had adhered and reached an appropriate density, they were washed three times with PBS, followed by fixation with 4% paraformaldehyde for 20 min. The cells were then treated with 0.5% Triton X-100 at room temperature for 20 min to facilitate antibody penetration. Subsequently, the cells were incubated with 5% goat serum for 30 min to block nonspecific binding and prevent background fluorescence interference.

### Statistical analysis

All data analyses for this study were conducted using GraphPad Prism 9.0.0 and R Studio 4.0.3. A *p*-value below 0.05 was deemed statistically significant.

## Approval of ethics and agreement to participate

The Ethics Committee of the Second Affiliated Hospital of Nanchang University approved all experiments conducted.

## Consent for publication

The publication was approved by all the authors.

## Data availability

The corresponding author can provide all data collected and analyzed in the current study upon request.

## Supporting information

This article contains supporting information.

## Conflict of interest

The authors declare that they have no conflicts of interest with the contents of this article.

## Supporting information

Spectrometry
